# Analysis of m6A-regulated genes and subtype classification in lupus nephritis

**DOI:** 10.1186/s12882-024-03549-3

**Published:** 2024-04-03

**Authors:** Diangeng Li, Yanchun Li, Kaiyi Zhu, Yuqing Yuan, Zheng He, Qianmei Sun, Meiling Jin

**Affiliations:** 1 Department of Nephrology, Beijing-Chaoyang Hospital, Capital Medical University, 100020 Beijing, China; 2 Department of Clinical Laboratory, Chinese PLA General Hospital, 100853 Beijing, China

**Keywords:** Lupus nephritis, m6A regulators, Bioinformatics analysis, Immunoinfiltration

## Abstract

**Background:**

Lupus nephritis (LN) is the most common and severe clinical manifestation of systemic lupus erythematosus (SLE). N6-methyladenosine (m6A) is a reversible RNA modification and has been implicated in various biological processes. However, the roles of m6A regulators in LN are not fully demonstrated.

**Methods:**

We downloaded the kidney tissue transcriptome dataset of LN patients and normal controls from the GEO database and extracted the expression levels of m6A regulators. We constructed and compared Random Forest (RF) and Support Vector Machine (SVM) models, and subsequently selected featured genes to develop nomogram models. The m6A subtypes were identified based on significantly differentially expressed m6A regulators, and the m6A gene subtypes were identified based on m6A-associated differential genes, and the two m6A modification patterns were comprehensively evaluated.

**Results:**

We obtained the GSE32591 and GSE112943 datasets from the GEO database, including 78 LN samples and 36 normal control samples. We extracted the expression levels of 20 m6A regulators. By RF analysis we identified 7 characteristic m6A regulators and constructed nomogramh models with these 7 genes. We identified two m6A subtypes based on these seven important m6A regulators, and the immune cell infiltration levels of the two subtype clusters were significantly different. We identified two more m6A gene subtypes based on m6A-associated DEGs. We calculated the m6A scores using the principal component analysis (PCA) algorithm and found that the m6A scores of m6A cluster A and gene cluster A were lower than those of m6A cluster B and gene cluster B. In addition, we found that the levels of inflammatory factors were also significantly different between m6A clusters and gene clusters.

**Conclusion:**

This study confirms that m6A regulators are involved in the LN process through different modes of action and provide new diagnostic and therapeutic targets for LN.

**Supplementary Information:**

The online version contains supplementary material available at 10.1186/s12882-024-03549-3.

## Introduction

Lupus nephritis (LN) is one of the most common and serious complications of systemic lupus erythematosus (SLE) with high morbidity and mortality rates [1]. The global annual incidence of SLE ranges from 1/100,000 to 8.7/100,000, and 40-60% of patients with SLE have LN at the time of onset. approximately 10-20% of patients with LN will eventually develop end-stage renal disease (ESRD) [2]. The treatment of LN is mainly based on glucocorticoids and immunosuppressive agents, but the therapeutic effect is not satisfactory [3]. Therefore, it is necessary to study the pathogenesis of LN in depth to propose new ideas for the diagnosis and treatment of LN and to improve the prognosis.

N6-methyladenosine (m6A) is one of the most prominent and abundant epigenetic modifications of mRNAs and lncRNAs in eukaryotic cells, and is a dynamic, reversible, and highly conserved process under the regulation of methyltransferases, demethylases, and binding proteins [4, 5]. The methylation of m6A is a process in which many proteins are involved. These proteins may broadly be divided into 3 categories: writer complexes, m6A demethylases, and function executions (readers) [6]. Previous studies have shown that m6A is widely involved in the development of multiple diseases, including a variety of tumors, systemic lupus erythematosus, etc. [7, 8]. Some studies suggest that m6A-related regulators in lupus nephritis (LN) are associated with the immune microenvironment, but the relationship between m6A and LN is not yet clear. Recent research indicates a strong correlation between m6A regulators in the kidney tissues of LN patients and activated NK cells, immune responses, and HLA genes. Additionally, seven m6A-related markers have been confirmed to be associated with the occurrence and progression of LN. Among these markers, CDC40 shows a positive correlation with glomerular filtration rate, suggesting a potential protective effect. On the other hand, CDC5L, HNrnbu, NUT21, PAPOLA, POLR2B, and WBP4 are negatively correlated with glomerular filtration rate, indicating that these genes may be involved in the kidney damage process in LN patients [9]. Abdelati AA et al. found that urinary CD14 monocytes could serve as a biomarker for diagnosing LN [10]. Some studies have confirmed that IGFBP2 is a promising biomarker for systemic lupus erythematosus (SLE) and LN [11]. However, the role of m6A regulators in LN needs to be further investigated.

In this study, we comprehensively evaluated the effects of m6A regulatory factors on the occurrence and subtype classification of LN. We assessed the correlation between m6A regulatory factors and the risk of LN by screening the important m6A regulatory factors and constructing a nomogram, and revealed the roles of different modes of m6A regulatory factors in LN, which provided a new theoretical basis for further research on m6A modification and new therapeutic strategies for LN.

## Materials and methods

### Data collection and preprocessing

Kidney tissue gene expression data from LN patients were obtained from the Gene Expression Omnibus (GEO) database in the GSE32591 and GSE112943 datasets, including data from 78 LN patients and 36 healthy controls. All datasets were preprocessed by the R packages “impute” (version 1.76) and “limma” (version 3.58.1). The datasets were then merged and processed to eliminate batch effects and analyzed using the R packages “limma” (version 3.58.1) and “sva” (version 3.50.0). Each sample was annotated as “LN” or “con” to distinguish LN samples from normal samples.

### Extraction and differential analysis of M6A regulators

Previous studies identified 26 m6A regulators, i.e., 9 writers, 15 readers, and 2 erasers, as shown in Table S1. We screened and visualized the m6A-regulated genes that were differentially overexpressed between LN and normal control samples. We used the “ggpubr” package (version 0.6.0) to draw box plots and the “pheatmap” package (version 1.0.12) to draw heat maps. We used the Perl language to map each extracted m6A gene to its chromosomal location and visualized it with the “RCircos” package (version 1.2.0).

### Random forest (RF) and support vector machine (SVM) model construction

The “Randomforest” package (version 4.7–1.1) was used to construct the RF model by randomly generating a large number of classification trees and iteratively scoring the classification results of the m6A regulators for each tree to obtain the classification results. The classification results of all individual trees were evaluated together and the “Caret” package (version 6.0–94) was used to rank the importance of the m6A regulators in the RF model. SVM models were constructed using the R software e1071 package (version 1.7–14) to find the optimal classification superplatform using the hub genes of the selected modules as independent variables. We assessed the models by generating classification reports (including Precision, Recall, F1-score)for the Random Forest (RF) and Support Vector Machine (SVM) models. We performed ROC analysis using the pROC package (version 1.7.2) to determine the predictive accuracy of the two models and calculated the area under the curve (AUC).

### Construction and validation of predictive nomogram

Based on the expression levels of important m6A regulators, nomogram was generated by the “rms” package (version 6.7-1), and Decision Curve Analysis (DCA) was performed to evaluate the predictive ability of the nomogram.

### Unsupervised cluster analysis

Based on the expression profiles of m6A regulators, we performed unsupervised cluster analysis of LN patient samples using the “ConsensusClusterPlus” package (version 1.66) and divided the samples into two m6A clusters. We also performed principal component analysis (PCA) to evaluate the classification, and single-sample gene set enrichment analysis (ssGSEA) to calculate the abundance of immune cells in LN samples.

### Identification of m6A subtypes based on DEG between m6A subtypes

We screened the differentially expressed genes (DEGs) between different m6A clusters using the “limma” software package (version 3.58.1), and performed GO (Gene Ontology) and KEGG (Kyoto Encyclopedia of Genes and Genomes) functional enrichment analyses using “clusterProfiler”. Functional enrichment analysis was performed using “clusterProfiler” (version 4.10.0). Sankey was plotted by “ggalluvial” (version 0.12.5), “ggplot2” (version 3.5.0) and “dplyr” (version 1.1.4).

### Calculation of the m6A score

To quantify the m6A patterns, we utilized the Principal Component Analysis algorithm to calculate the m6A score for each sample. Initially, Principal Component Analysis (PCA) was employed to differentiate the m6A patterns. Subsequently, the m6A score was computed using the formula: m6A score = PC1i, where PC1 denotes the first principal component, and i represents the expression of Differentially Expressed Genes (DEGs).

### Statistical analysis

All analyses were performed in R software (version 4.2.2). *p* < 0.05 was considered statistically significant.

## Results

### Profile of m6A regulators in lupus nephritis

**Fig. 1:**
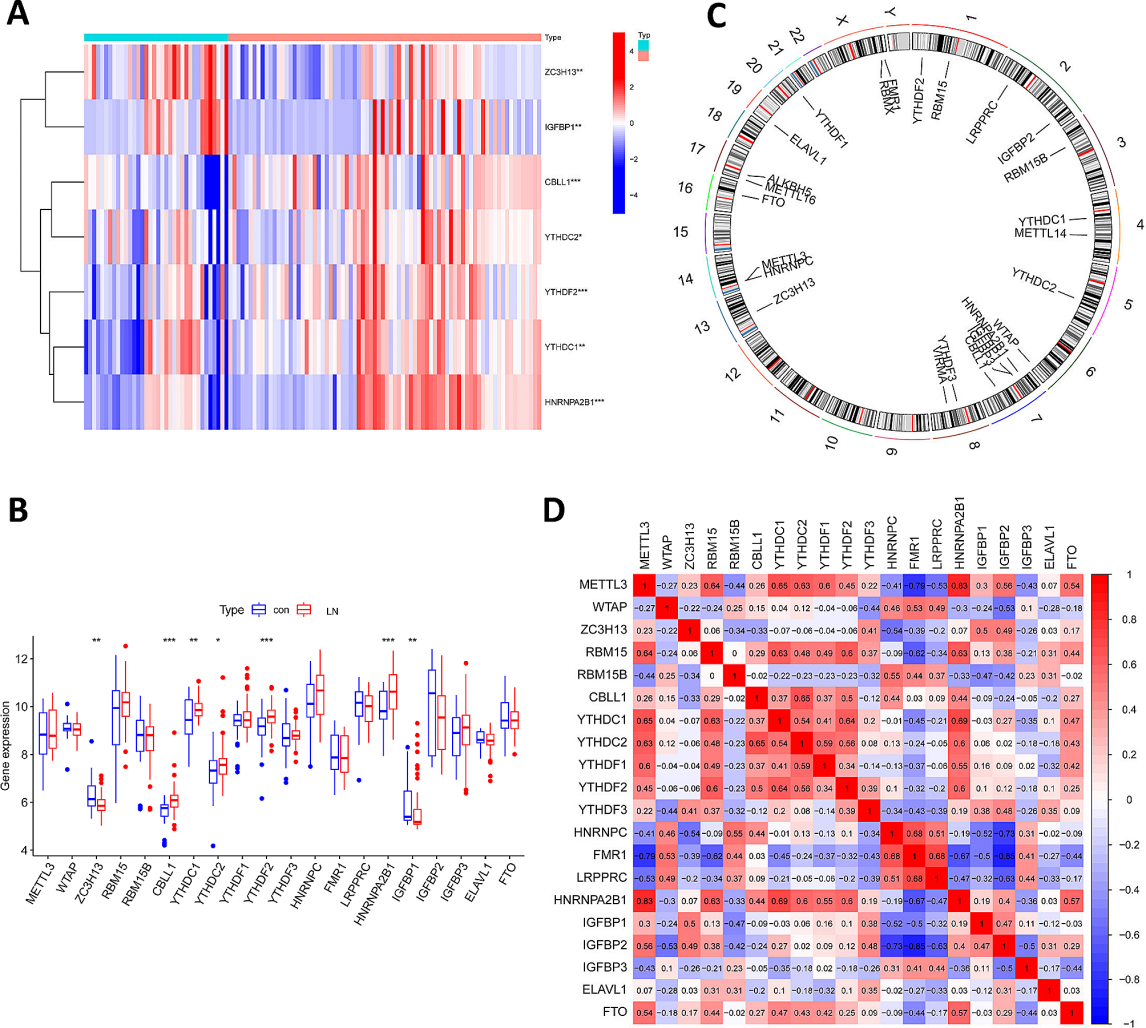
Landscape of and correlations between m6A-regulated genes in LN. **A** Heatmap of the expression of m6A regulatory factors in control and LN; **B** Histogram of the expression of m6A regulatory factors in control and LN; **C** Chromosomal location of m6A regulatory factors. **D** The heatmap of the correlations between m6A regulatory factors. * *p* < 0.05, ** *p* < 0.01, *** *p* < 0.001

We identified a total of 4739 Differentially Expressed Genes (DEGs) between control and lupus nephritis (LN) samples, and further analyzed the differential expression levels of m6A regulator. We found that ZC3H13 and IGFBP1 were lowly expressed in LN, and CBLL1, YTHDC1, YTHDC2, YTHDF2 and HNRNPA2B1 were highly expressed in LN (Fig. 1A and B). The chromosomal locations of m6A regulators were visualized using the “RCircos” package (Fig. 1C). We also explored the m6A regulators for correlation analysis, as shown in Fig. 1D.

### Construction of RF and SVM models

**Fig. 2:**
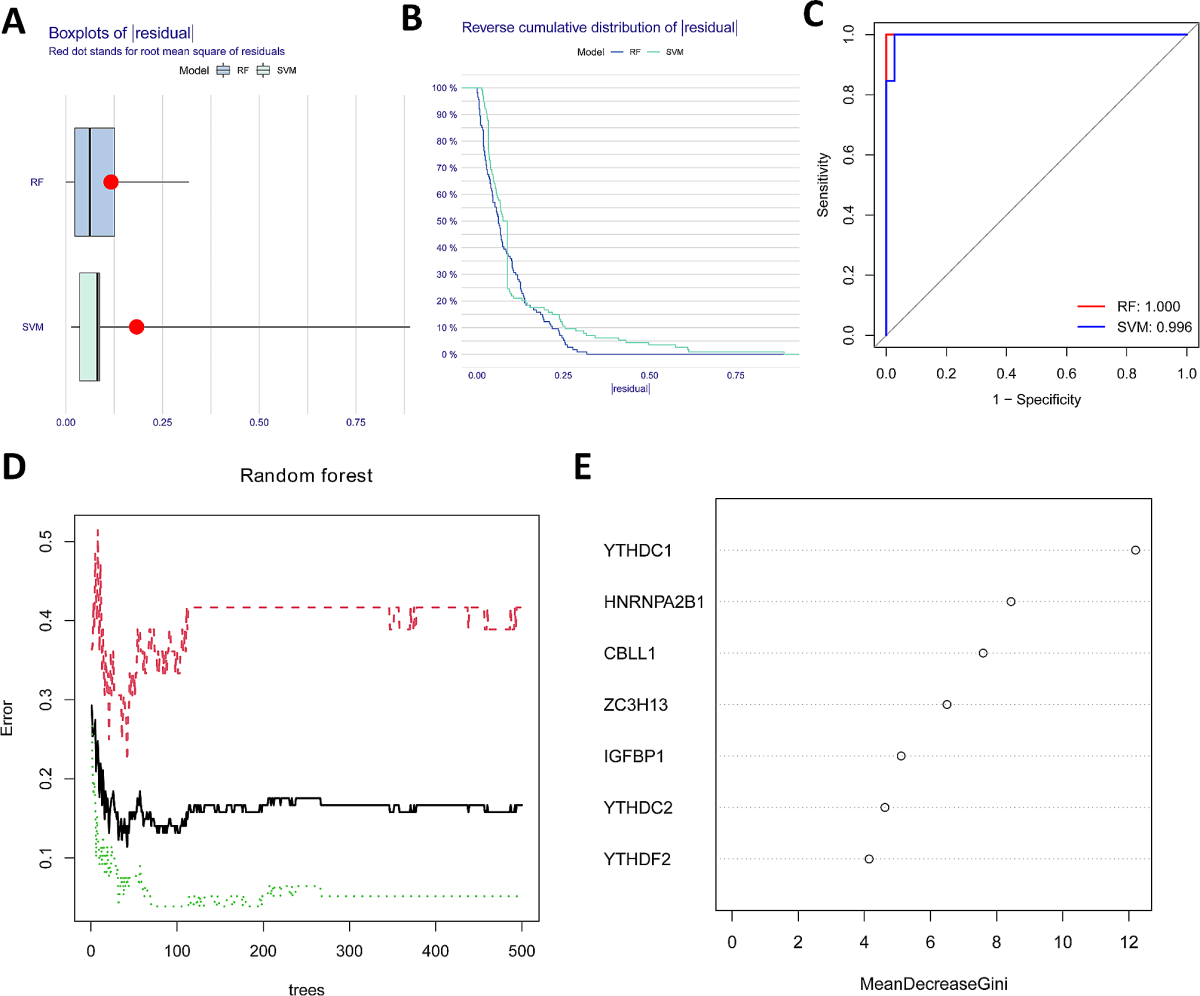
RF and SVM models construction: **A** The boxplot of residuals for the RF model and the support vector machine (SVM) model; **B** The inverse cumulative distribution of residuals for the RF model and the SVM model; **C** ROC curves for the RF and SVM models; **D** Random forest plot; **E** The importance of the 14 differentially expressed m6A regulators in the RF model

We constructed RF and SVM models and the candidate m6A modulators were selected to predict the occurrence of LN. In the RF model, the precision value was 1, the recall value was 1, and the F1 value was 1. In the SVM model, the precision value was 0.987(0.963,1.012), the recall value was 1.000(1.000,1.000), and the F1 value was 0.993. The residual box plots and the inverse cumulative distribution of residuals showed that the RF model had the smallest residuals (Fig. 2A and B), and the AUC values of the ROC curves also showed that the RF model had higher accuracy than the SVM model (Fig. 2C), so we chose the RF model as the best model for predicting LN occurrence. The LN signature genes were screened by RF, and YTHDC1, HNRNPA2B1, CBLL1, ZC3H13, IGFBP1, YTHDC2, and YTHDF2 were selected as candidate genes (Fig. 2D and E).

### Construction of the nomogram model

**Fig. 3:**
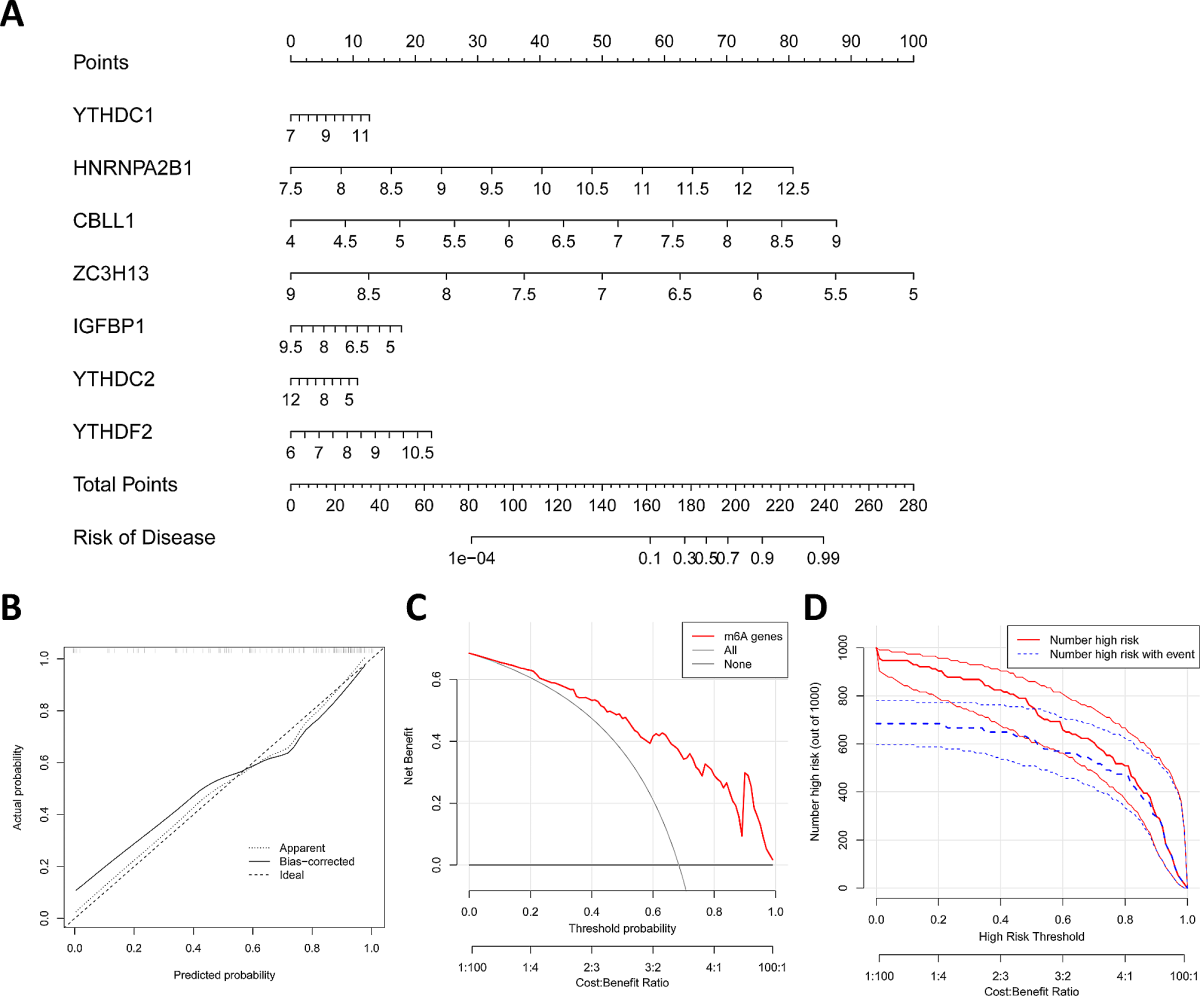
Construction of the nomogram model: **A** Construction of the nomogram model based on seven m6A regulator candidate genes; **B** Calibration curves showing the predictive ability of the nomogram model; **C** Decision curves showing the decision-making ability of the nomogram model; **D** Clinical impact curves assessing the clinical impact of the nomogram model

We used the “rms” package in R to construct a nomogram model based on these 7 important m6A regulators to predict the prevalence of LN (Fig. 3A). The calibration curves showed that the predictions of the nomogram models were accurate (Fig. 3B). Decision curves showed that decisions based on the nomogram model favored LN (Fig. 3C). The clinical impact curve showed that the predictive power of the nomogram model was significant (Fig. 3D).

### Identification of different m6A patterns

**Fig. 4:**
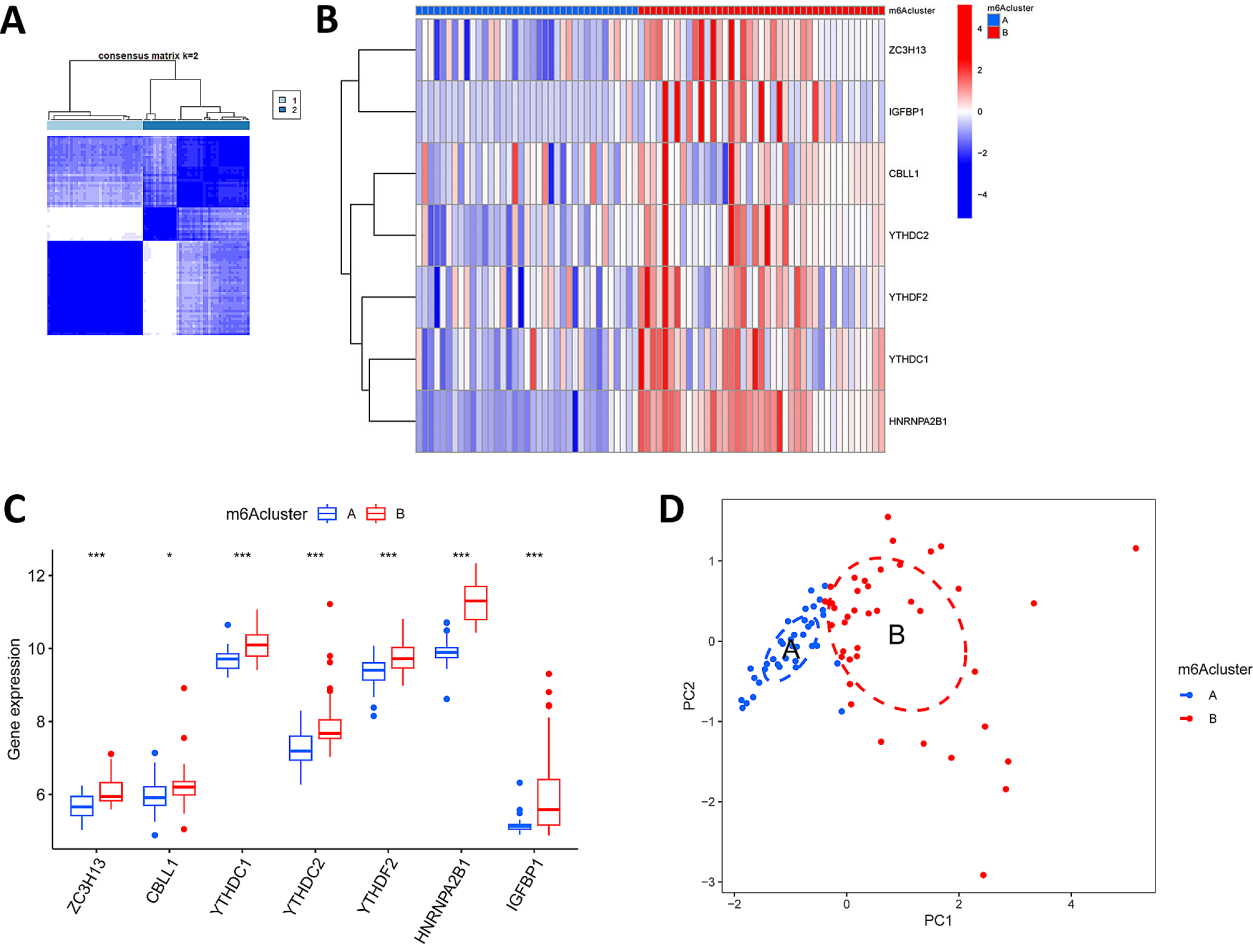
Clustering analysis of 7 significant m6A regulators associated with LN. **A** When k = 2, the consensus clustering analysis was the most reliable. **B** The heatmap of the differential expression of the 7 m6A-regulated genes between cluster A and cluster B. **C** The box plot of the differential expression of the 7 m6A regulators between cluster A and cluster. **D** Principal component analysis (PCA) of cluster A and cluster B. ***P < 0.001

Using the “ConsensusClusterPlus” package in R, a consensus clustering method was used to identify different m6A patterns based on 7 important m6A regulators, and 2 m6A patterns (cluster A, cluster B) were identified (Fig. 4A). Cluster A contained 37 cases and cluster B contained 41. heatmaps and histograms suggested that YTHDC1, HNRNPA2B1, CBLL1, ZC3H13, IGFBP1, YTHDC2, and YTHDF2 were more highly expressed in cluster B than in cluster A (Fig. 4B and C). PCA showed that 7 important m6A regulators could distinguish between these two m6A patterns (Fig. 4D).

**Fig. 5:**
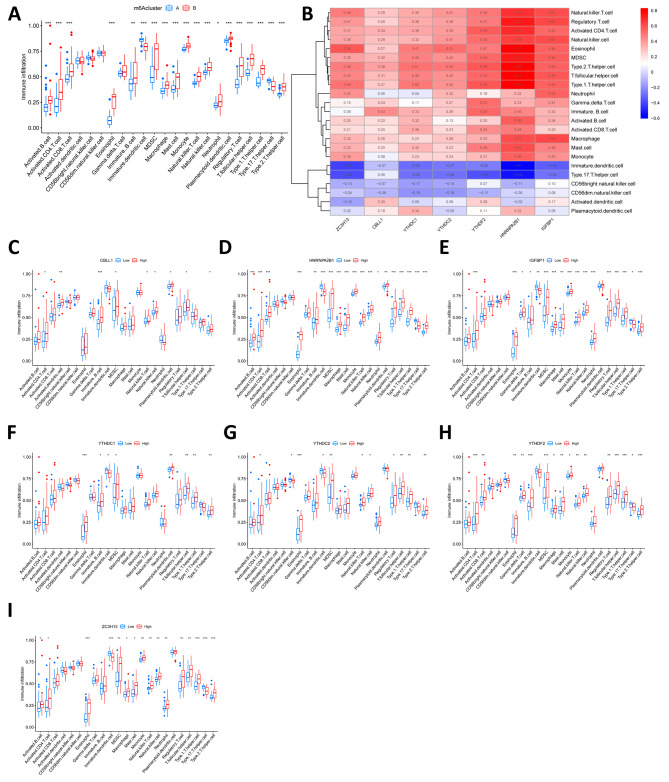
Single-sample gene set enrichment analysis (ssGSEA). **A** Differences in immune cell infiltration between cluster A and cluster B. **B** Immuno-correlation analysis between m6A-regulated genes and immune cells. **C-I** Differential immune cell infiltration between groups with lower and higher expression of these 7 m6A-regulated genes

We applied ssGSEA to calculate the abundance of immune cells in LN samples and assessed the correlation between the seven important m6A regulators and immune cells, and we found that most immune cells were significantly more infiltrated in cluster B than in cluster A (Fig. 5A). We performed a correlation analysis between m6A regulatory genes and immune cells and found that these seven important m6A regulators were positively correlated with most immune cells (Fig. 5B). As shown in Fig. 5C-I, the expression of m6A regulators also affected the degree of immune cell infiltration.

### Construction of the m6A gene signature

**Fig. 6:**
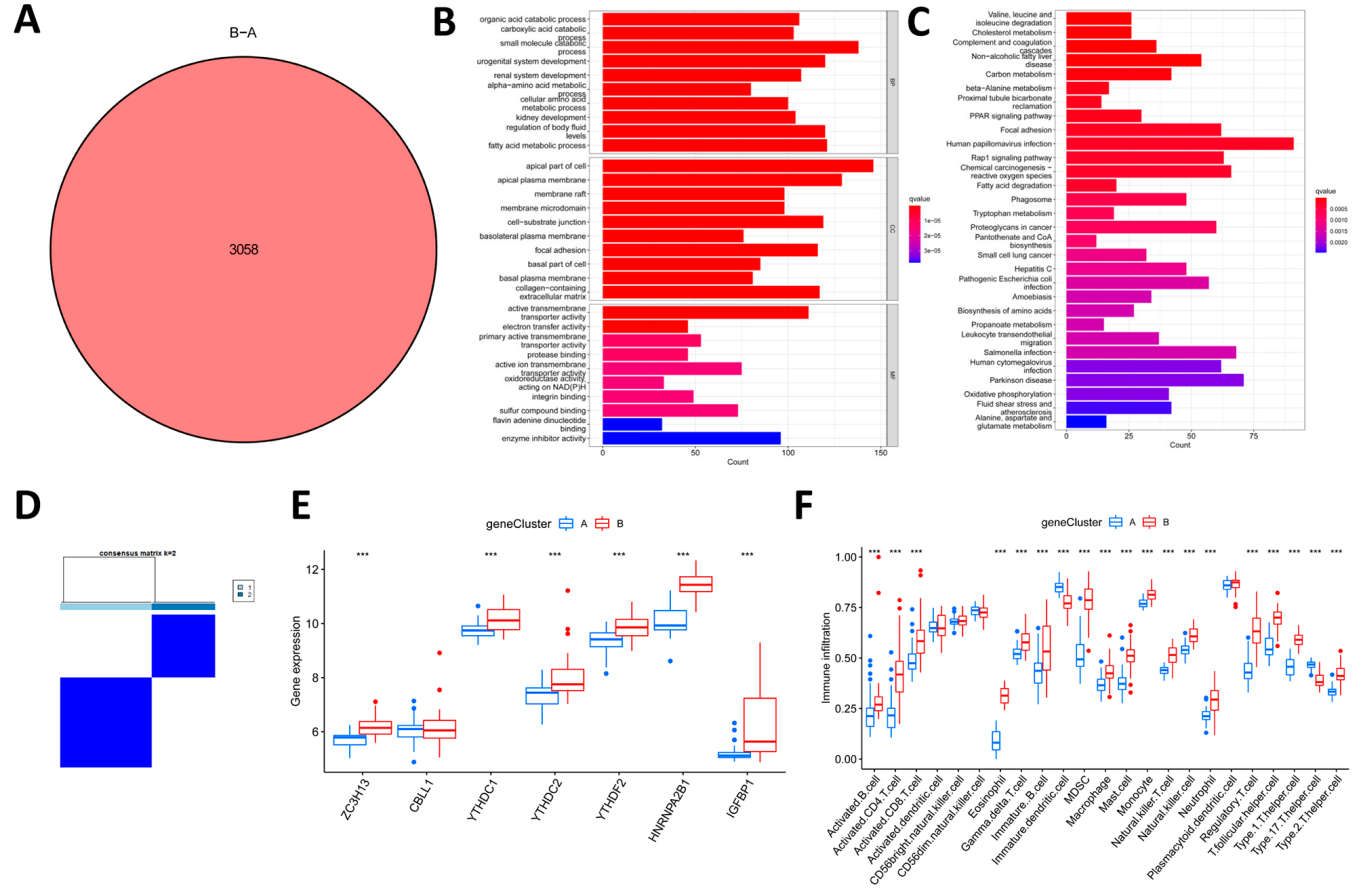
Consensus clustering analysis of the differentially expressed genes (DEGs). **A** A total of 3058 DEGs were identified between cluster A and cluster B. **B** Gene Ontology analysis of these DEGs. **C** Kyoto Encyclopedia of Genes and Genomes pathway analysis of these DEGs. **D** When k = 2, the consensus clustering analysis was the most reliable. **E** The box plot of the differential expression of the 7 m6A regulators between cluster A and cluster B. **F** Differences in immune cell infiltration between cluster **A** and cluster **B**

We screened the differentially expressed genes (DEGs) between different m6A clusters which were the m6A-associated DEGs, and identified 3058 m6A-associated DEGs between the two m6A subtypes (Fig. 6A). We analyzed these DEGs by GO and KEGG enrichment, and in biological processes (BP), DEGs were mainly involved in the organic acid catabolic process, carboxylic acid catabolic process, small molecule catabolic process, etc. In the cellular component (CC), DEG is mainly enriched in apical part of cell, apical plasma membrane, membrane raft, etc. In molecular function (MF), DEG is mainly associated with the apical part of cell, apical plasma membrane, and membrane raft. The molecular function (MF) was mainly related to active transmembrane transporter activity, electron transfer activity, primary active transmembrane transporter activity, etc. (Fig. 6B). KEGG analysis showed that DEG was mainly involved in Valine, leucine and isoleucine degradation, Cholesterol metabolism, Complement and coagulation cascades (Fig. 6C). We categorized patients according to DEG into gene cluster A and cluster B by consensus clustering method (Fig. 6D). YTHDC1, HNRNPA2B1, ZC3H13, IGFBP1, YTHDC2, and YTHDF2 were significantly higher expressed in gene cluster B than in gene cluster A (Fig. 6E). ssGSEA analysis revealed that the infiltration of the majority of the immune cells in gene cluster B was significantly higher than gene cluster A (Fig. 6F).

**Fig. 7:**
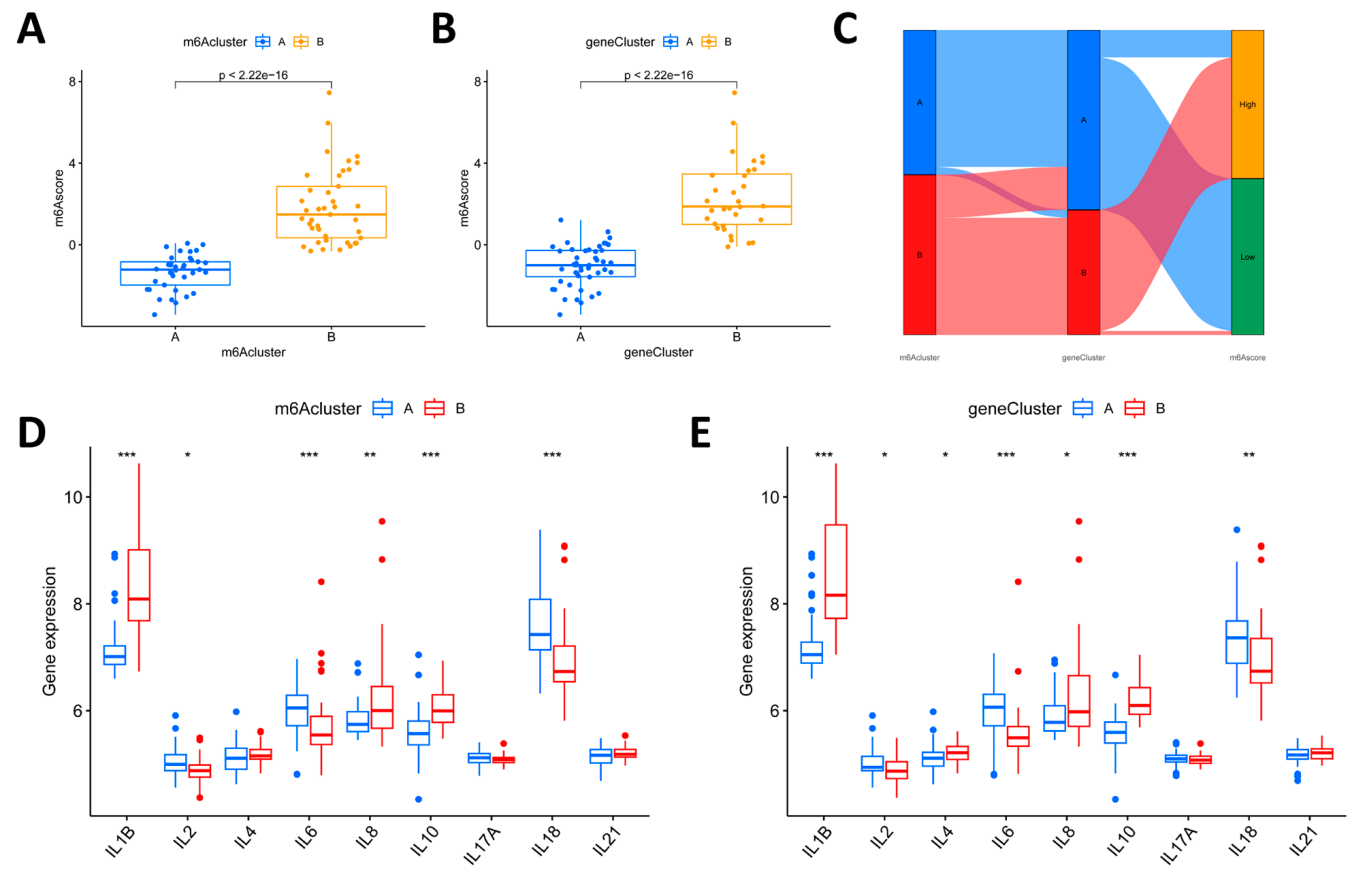
Role of m6A subtypes and m6A gene subtypes in distinguishing LN. **A** m6A score differences between m6A subtypes. **B** A m6A score differences between m6A gene subtypes. **C** Sankey diagram displaying the distribution of LN patients in the two m6A clusters, two gene clusters and two m6A score groups. **D** The expression levels of inflammatory factors in the two m6A clusters. **E** The expression levels of inflammatory factors in the two gene clusters. ***P < 0.001

We scored LN patients based on the expression of m6A regulators using principal component analysis (PCA), defined as m6A scores. m6A scores between m6A clusters and gene clusters differed significantly (Fig. 7A and B). Sankey plots showed the distributions of the two m6A clusters, two gene clusters, and two m6A scores. m6A Cluster A and Gene Cluster A corresponded to low m6A scores, while m6A cluster B and gene cluster B corresponded to high m6A scores (Fig. 7C). As shown in Fig. 7D and E, there were also significant differences in inflammatory factor levels between m6A clusters and between gene clusters. Therefore, the two subgroups associated with m6A (m6A clusters and m6A gene clusters) can help us to predict inflammatory factor levels and disease risk scores in LN patients.

## Discussion

Lupus nephritis is the leading cause of morbidity and mortality in SLE, and many patients end up with chronic kidney disease or ESRD due to limited drug therapy [12, 13]. Therefore, it is imperative to gain a deeper understanding of the mechanisms of LN and explore potential therapeutic targets to improve the prognosis of LN patients. It has been found that m6A regulators are involved in the development of many human diseases, and the overall role of m6A regulators in LN has not been fully recognized [14]. Therefore, identifying m6A modification patterns in LN will help enhance our understanding of the pathogenesis of the disease and explore new therapeutic strategies.

In this study, we comprehensively investigated the m6A modification characteristics of LN patients. m6A regulators were significantly different in expression levels between normal controls and LN patients, and we found that ZC3H13 and IGFBP1 were lowly expressed in LN, and CBLL1, YTHDC1, YTHDC2, YTHDF2, and HNRNPA2B1 were highly expressed in LN, which suggests that m6A modification may be closely related to the development of LN. It was found that SLE patients showed reduced expression of natural autoantibodies including IGFBP1, which may increase the susceptibility to SLE [15]. YTHDC2 is involved in the process of diabetic nephropathy [16]. The logistic regression analysis revealed that decreased mRNA expression of YTHDF2 was a risk factor for SLE [17]. The role of m6A regulators in LN remains to be further investigated. In this study, we identified alterations and interactions of m6A regulators in LN at the transcriptional level. To construct m6A regulatory factor-related prediction models, we compared the random forest (RF) and support vector machine (SVM) models. RF is a learning algorithm that combines different decision trees. The RF model consists of independent decision trees, each of which is generated based on random samples, and each of which learns and predicts independently, with the final result being based on the average of all trees [18]. SVM is a discriminative classifier that uses classification hyperplanes to define the classification. The model is trained using labeled training samples and then the optimal hyperplane output is used to classify the test samples [19]. By comparing the residuals and AUC values, RF model was more suitable as a training model. Therefore, we chose the RF model as the best model for predicting LN occurrence, and screened the LN signature genes by RF, and YTHDC1, HNRNPA2B1, CBLL1, ZC3H13, IGFBP1, YTHDC2, and YTHDF2 were selected as candidate genes. These seven most important genes were included in the construction of the LN residual maps.

To assess the characterization of m6A regulatory patterns in the classification of immune profiles, we grouped all LN patients into two subgroups by consensus clustering analysis. We applied ssGSEA to calculate the abundance of immune cells in LN samples and assessed the correlation between seven important m6A modulators and immune cells, and found that the majority of immune cells were significantly more infiltrated in cluster B than in cluster A, and that the m6A modulators were positively correlated with the majority of immune cells. We found that T cell, macrophage, and monocyte infiltration was higher in cluster B than in cluster A. SLE is characterized by dysregulation of autoreactive B cells and many other types of immune cells, including myeloid cells [20]. T cells are central to the pathogenesis of lupus nephritis (LN), and blockade of CD6 in a model of spontaneous lupus and immune-complex glomerulonephritis resulted in significant decreases in immune cells, inflammatory markers, and disease markers [21]. Macrophages involved in the LN can be either tissue-resident or monocyte-derived macrophages.CD16 + monocytes are recruited to the inflamed kidney via CCL2, CSF-1, fractalkine, and bone bridging proteins and differentiate into macrophages under microenvironmental influence. Renal macrophages are heterogeneous with pro-inflammatory and anti-for functions, which, due to imbalance in the LN, lead to chronic inflammation, fibrosis, and renal hypoplasia. Targeted therapy against monocytes and/or macrophages may become a new approach for LN treatment [22, 23]. We identified 3058 m6A-associated DEGs between LN and non-LN patients and then performed unsupervised cluster analysis to categorize patients into two gene clusters. We found that the m6A clusters and gene clusters had similar trends in immune cell infiltration levels, which helped predict the clinical features of LN. We quantified m6A subtypes by calculating m6A scores, and both m6A cluster A and gene cluster A had lower m6A scores. Therefore, m6A modifiers can not only distinguish LN from normal individuals, but also predict the prognosis of LN patients based on m6A subtyping for further molecular typing. In addition, there were significant differences in the levels of inflammatory factors between m6A clusters and gene clusters.

However, this study still has some limitations. Our conclusions were drawn from the analysis of datasets in a public database, and there is a lack of multiple clinical datasets and experimental studies to validate the relationship between m6A regulators and LN. We will add large clinical cohorts for validation and conduct relevant mechanistic studies in subsequent studies.

In conclusion, we performed a comprehensive analysis of m6A regulatory factors in patients with LN, suggesting regulatory mechanisms affecting immune cell infiltration characteristics and inflammatory factor levels, and constructed nomogram to determine their value in predicting LN risk. Two m6A subtypes were identified based on the expression of seven important m6A regulators, and m6A cluster B versus gene cluster B may be significantly associated with LN.

### Electronic supplementary material

Below is the link to the electronic supplementary material.

Supplementary Material 1

## Data Availability

The original datasets were downloaded from the Gene Expression Omnibus (https://www.ncbi.nlm.nih.gov/geo/). The datasets used and/or analyzed during the current study available from the corresponding author on reasonable request.

## Abbreviations

LN: Lupus nephritis
SLE: Systemic lupus erythematosus
RF: Random Forest
SVM: Support Vector Machine
PCA: Principal component analysis
ESRD: End-stage renal disease
DEGs: Differentially expressed genes
GO: Gene Ontology
KEGG: Kyoto Encyclopedia of Genes and Genomes
